# P-689. Respiratory Syncytial Virus Infection in Patients with Cancer at Referral Oncologic Center in Mexico City

**DOI:** 10.1093/ofid/ofae631.885

**Published:** 2025-01-29

**Authors:** Jack N Salto-Quintana, Alex Cardona Ortiz, Nancy Martinez-Rivera, Eduardo Becerril-Vargas, Alexandra Martin-Onräet, Diana Vilar-Compte

**Affiliations:** Instituto Nacional de Cancerologia, Mexico City, Distrito Federal, Mexico; Instituto Nacional de Cancerología, Mexico City, Distrito Federal, Mexico; Hospital General Manuel Gea Gonzalez, Mexico City, Distrito Federal, Mexico; Instituto Nacional de Enfermedades Respiratorias, Mexico City, Distrito Federal, Mexico; Instituto Nacional de Cancerologia, Mexico City, Distrito Federal, Mexico; Instituto Nacional de Cancerologia, Mexico City, Distrito Federal, Mexico

## Abstract

**Background:**

Respiratory Syncytial Virus (RSV) infection has a high risk of complications in patients with cancer. This study aimed to describe the clinical characteristics and outcomes of oncologic patients infected with RSV in a tertiary care hospital.

**Methods:**

A retrospective study was conducted over ten years at the National Cancer Institute in Mexico City. Adults with a confirmed diagnosis of cancer and RSV by polymerase chain reaction (PCR) were included.

**Results:**

Fifty-seven cases of RSV infection were included, 29 (50.9%) were women, the median age was 48 years (IQR 34-12), 15 (26.3%) were > 60 years; 36 (61%) had hematologic malignancies, and 23 (38.9%) solid tumors (two patients had both); 31 (54.4%) patients had active cancer; 24 (42.1%) received chemotherapy in the preceding 30 days. The most frequent comorbidities were hypertension (28.1%), obesity (17.5%), and diabetes (14%). Twenty-four (42.1%) patients had > 5 points on the Charlson comorbidity index. Fifty-seven percent of patients presented Systemic Inflammatory Response Syndrome.

Fifty-four percent were diagnosed during winter (December to February) (n=22). According to the immunodeficiency index for progression risk prediction in hematological patients with RSV disease, 13 (36.1%) patients were at high risk.

Seventy percent were hospitalized, although only 52.6% for pneumonia. Respiratory progression or deterioration was documented in 11 (19.3%) patients; 5 (8.8%) required mechanical ventilation, and 4 (7%) were admitted to the ICU.

Eleven (19.3%) patients were treated with ribavirin, with no outcome differences (P= NS). Co-infection was documented in 15 patients (26.3%): 4 (7%) had bacteremia, 2 (3.5%) had bacterial pneumonia, and 5 (8.7%) had other bacterial infections. Seven (12.2%) patients had viral co-infection: 4 (7%) with COVID-19, 2 (3.5%) with rhinovirus and one (1.7%) with influenza. The 30-day mortality was 14.8% (n=8/54).

**Conclusion:**

In this cohort, morbidity and mortality were high. The therapeutic options for RSV infection are limited. Currently, there are effective preventive measures for some risk groups; however, they do not include oncologic patients. These data provide relevant information about the burden of disease in adults with cancer.

**Disclosures:**

All Authors: No reported disclosures

